# Antihyperlipidemic and antiperoxidative effect of Diasulin, a polyherbal formulation in alloxan induced hyperglycemic rats

**DOI:** 10.1186/1472-6882-5-14

**Published:** 2005-06-22

**Authors:** Ramalingam Saravanan, Leelavinothan Pari

**Affiliations:** 1 Department of Biochemistry, Faculty of Science, Annamalai University, Annamalai Nagar, Tamil Nadu – 608 002, India

## Abstract

**Background:**

This study was undertaken to investigation the effect of Diasulin, a poly herbal drug composed of ethanolic extract of ten medicinal plants on blood glucose, plasma insulin, tissue lipid profile, and lipidperoxidation in alloxan induced diabetes.

**Methods:**

Ethanolic extract of Diasulin a, poly herbal drug was administered orally (200 mg/kg body weight) for 30 days. The different doses of Diasulin on blood glucose and plasma insulin in diabetic rats were studied and the levels of lipid peroxides [TBARS, and Hydroperoxide] and tissue lipids [cholesterol, triglyceride, phospholipides and free fatty acids] were also estimated in alloxan induced diabetic rats. The effects were compared with glibenclamide.

**Result:**

Treatment with Diasulin and glibenclamide resulted in a significant reduction of blood glucose and increase in plasma insulin. Diasulin also resulted in a significant decrease in tissue lipids and lipid peroxide formation. The effect produced by Diasulin was comparable with that of glibenclamide.

**Conclusion:**

The decreased lipid peroxides and tissue lipids clearly showed the antihyperlipidemic and antiperoxidative effect of Diasulin apart from its antidiabetic effect.

## Background

Diabetes mellitus is syndrome, initially characterized by a loss of glucose homeostasis resulting from defects in insulin secretion, insulin action both resulting impaired metabolism of glucose and other energy- yielding fuels such as lipids and protein [1]. Experimental diabetes in animals has provided considerable insight into the physiologic and biochemical derangement of the diabetic state. Many of the derangement have been characterized in hyperglycemic animals. Significant changes in lipid metabolism and structure also occur in diabetes [2]. In these cases the structural changes are clearly oxidative in nature and are associated with development of vascular disease in diabetes [3]. In diabetic rats, increased lipidperoxidation was also associated with hyperlipidemia [4]. Liver, an insulin dependent tissue that plays a pivotal role in glucose and lipid homeostasis and it is severely affected during diabetes [5]. Liver and kidney participates in the uptake, oxidation and metabolic conversion of free fatty acids, synthesis of cholesterol, phospholipids, and triglycerides. During diabetes, a profound alteration in the concentration and composition of lipids occurs.

Despite the great strides that have been made in the understanding and management of diabetes, the disease and disease related complications are increasing unabated [6]. Inspite of the presence of known antidiabetic medicine in the pharmaceutical market, remedies from medicinal plants are used with success to treat this disease [7]. Many traditional plant treatments for diabetes are used throughout the world. Plant drugs [8] and herbal formulation [9-11] are frequently considered to be less toxic and more free from side effects than synthetic one. Based on the WHO recommendations hypoglycemic agents of plant origin used in traditional medicine are important [12]. The attributed antihyperglycemic effects of these plants is due to their ability to restore the function of pancreatic tissues by causing an increase in insulin output or inhibit the intestinal absorption of glucose or to the facilitation of metabolites in insulin dependent processes. Hence treatment with herbal drugs has an effect on protecting β-cells and smoothing out fluctuation in glucose levels [13,14]. In general, there is very little biological knowledge on the specific modes of action in the treatment of diabetes, but most of the plants have been found to contain substances like glycosides, alkaloids, terpenoids, flavonoids etc., that are frequently implicated as having antidiabetic effects [15]. In the traditional system of Indian medicine, plant formulation and combined extracts of plants are used as drug of choice rather than individual. Various herbal formulations such as diamed [16], coagent db [17] and hyponidd [18], are well known for their antidiabetic effects. Diasulin is a poly herbal drug composed of ten medicinal plants (Table 1), which are already reported for their antidiabetic activity. In our previous study, we have demonstrated the antidiabetic effect of Diasulin in alloxan diabetic rats [19]. The present investigation was undertaken to study the effect of the potential antidiabetic herbal formulation, Diasulin on lipidperoxidation and tissue lipid profile in alloxan diabetic rats. The effects produced by this drug on different parameters were compared with glibenclamide, a reference drug.

**Table 1:** Diasulin (Composition and concentration)

**SI. No**	**Botanical name**	**Common name**	**Family**	**Part used**	**Concentration (mg/dL)**
1	*Cassia auriculata*	Tanner's cassia	**Ceasalpinaceae**	Flower	40
2	*Coccinia indica*	Little gourd	**Cucurbitaceae**	Fruit	40
3	*Curcuma longa*	Turmeric	**Zingiberaceae**	Rhizome	40
4	*Emblica officinalis*	Indian gooseberry	**Euphorbiaceae**	Fruit	20
5	*Gymnema sylvestre*	Ram's horn	**Asclepiadaceae**	Leaves	20
6	*Momordica charantia*	Bitter gourd	**Cucurbitaceae**	Fruit	30
7	*Scoparia dulcis*	Sweet broom weed	**Scrophulariaceae**	Whole Plant	40
8	*Syzigium cumini*	Jamun	**Myrtaceae**	Seed	20
9	*Tinospora cardifolia*	Gulancha tinospora	**Menispermaceae**	Root	20
10	*Trigonella foenum graecum*	Fenugreek	**Fabaceae**	Seed	50

## Methods

### Experimental animals

Male Wistar rats of body wt. 180–200 g were obtained from central Animal House, Raja Muthiah Medical College, Annamalai University. The animals were fed on standard pellet diet (Hindustan Lever, Mumbai, India) and water ad libitum. The rats used in the present study were maintained in accordance with guidelines of the National Institute of Nutrition, Indian Council of Medical Research, Hyderbad, India and the study approved by the ethical committee (Vide No: 88, 2002).

### Drug and chemicals

Ethanolic extract of ten medicinal plants, which are involved in preparation of Diasulin, was a gift from herbal remedies, Pondicherry, India.

The residual extracts of the antidiabetic plants were mixed and named as Diasulin was prepared (Table 1) on the basis of an ayurvedic antidiabetic formulation proposed by Pandey et al. (1995) [20]. 500 g of each plant (chopped into small pieces) was extracted individually were, soaked overnight in 1.5 litres of 95% ethanol. This suspension was filtered and the residue was resuspended in an equal volume of 95% ethanol for 48 h and filtered again. The two filtrates were pooled and the solvents were evaporated in a rotavapor at 40° – 50°C under reduced pressure and lyophilized. Alloxan monohydrate was purchased from BDH Chemicals, Poole, England. Enzyme linked immunosorbant assay (ELISA) kit for insulin assay was purchased from Boehringer Mannheim, Germany. All other biochemicals used in this experiment were purchased from Sigma Chemical Company Inc., St Louis, Mo, and USA. The chemicals were analytical grade.

### Drug administration

Diasulin was suspended in distilled water and administered orally through intragastric tube at the following doses of 50, 100 and 200-mg/kg body weight.

### Experimental induction of diabetes in rats

The rats were injected intraperitoneally with alloxan monohydrate dissolved in sterile normal saline at a dose of 150 mg/kg body wt [21]. After 2 weeks, rats with moderate diabetes having glycosuria (indicated by Benedict's qualitative test) and hyperglycemia (i.e. with a blood glucose of 200–300 mg/dl) were used for the experiment.

### Experimental design

In the experiment, a total of 42 rats (30 diabetic surviving rats, 12 normal rats) were used. The rats were divided into seven groups of six rats each after the induction of alloxan diabetes. Group1: Normal treated rats. Group2: Normal rats given aqueous solution of Diasulin (200 mg/kg body weight) daily using an intragastric tube for 30 days. Group 3: Diabetic control rats. Group 4: Diabetic rats given aqueous solution of Diasulin (50 mg /kg body weight) daily using an intragastric tube for 30 days. Group 5: Diabetic rats given aqueous solution of Diasulin (100 mg /kg body weight) daily using an intragastric tube for 30 days. Group 6: Diabetic rats given aqueous solution of Diasulin (200 mg /kg body weight) daily using an intragastric tube for 30 days. Group 7: Diabetic rats given aqueous solution of glibenclamide (600 μg/kg body weight) daily using an intragastric tube for 30 days.

At the end of 30 days, all the rats were killed by decapitation under pentobarbitone sodium (60 mg/kg) anaesthesia. Blood was collected in tubes containing potassium oxalate and sodium fluoride solution for the estimation of blood glucose and plasma was separated for the assay of insulin. Liver and kidney were immediately dissected out, washed in ice-cold saline to remove the blood. The tissues were weighed and 10% tissue homogenate was prepared with 0.025 M Tris – Hcl buffer, pH 7.5. After centrifugation at 200 rpm for 10 min, the clear supernatant was used to measure thiobarbituric acid reactive substances (TBARS) and hydroperoxides. For the determinations of lipids the liver and kidney tissues were weighed and lipids were extracted from tissues by the method of Folch et al [22] using chloroform – methanol mixture (CHCl_3_: MeOH)(2:1 v/v).

## Biochemical analysis

### Estimation of blood glucose and plasma insulin

Blood glucose was determined by the O-toluidine method [23]. Plasma insulin was assayed by ELISA, using Boeheringer- Mannheim Kit with a Boeheringer analyser ES300.

### Estimation of lipid peroxidation

Lipid peroxidation in liver and kidney were estimated colorimetrically by thiobarbituric acid reactive substances TBARS and hydroperoxides by the method of Niehius and Samuelsson [24] and Jiang et al. [25], respectively. In brief, 0.1 ml of tissue homogenate (Tris-Hcl buffer, pH 7.5) was treated with 2 ml of (1:1:1 ratio) TBA-TCA-HCl reagent (thiobarbituric acid 0.37%, 0.25 N HCl and 15% TCA) and placed in water bath for 15 min, cooled. The absorbance of clear supernatant was measured against reference blank at 535 nm.

0.1 ml of tissue homogenate was treated with 0.9 ml of Fox reagent (88 mg butylated hydroxytoluene (BHT), 7.6 mg xylenol orange and 9.8 mg ammonium ion sulphate were added to 90 ml of methanol and 10 ml 250 mM sulphuric acid) and incubated at 37°C for 30 min. The color developed was read at 560 nm colorimetrically. Hydroperoxides was expressed as mM/100 g tissue.

### Estimation of lipids

Lipids were extracted from tissues by the method of Folch et al [22] using chloroform – methanol mixture (CHCl_3_: MeOH) (2:1 v/v). The total cholesterol was estimated by the method of zlatkis et al [26]. To 0.1 ml of the lipid extract, 9.9 ml of ferric chloride-acetic acid reagent was added and allowed to stand for 15 min and then centrifuged. To 5 ml of the supernatant, add 3 ml of Conc. H_2_So_4_. The colour developed was read after 20 min at 560 nm against a reagent blank. Values were expressed as mg/100 g tissue.

Triglycerides were estimated by the method of foster and Dunn [27]. To an aliquot of lipid extract, evaporated to dryness. 0.1 ml of methanol was added followed by 4 ml of isopropanol. 0.4 g of alumina was added to all the tubes and shaken well for 15 min. Centrifuged and then 2 ml of the supernatant was transferred to labeled tubes. The tubes were placed in a water bath at 65°C for 15 min for saponification after adding 0.6 ml of the saponification reagent followed by 0.5 ml of acetyl acetone reagent. After mixing, the tubes were kept in a water bath at 65°C for 1 h, the contents were cooled and absorbance was read at 420 nm. The triglyceride content was expressed as mg/100 g tissue.

Phospholipid content was determined by the method of Zilversmit et al [28]. To 0.1 ml of lipid extract, added 1 ml of 5 N H_2_SO_4 _and 1 ml of concentrated nitric acid and digested to a colourless solution. The phosphorus content in the extract was determined by the method of Fiske and Subba Row [29]. The values were expressed as g/100 g tissue.

Free fatty acids were estimated by the method of Falholt et al [30]. 0.1 ml of lipid extract was evaporated to dryness. 1 ml of phosphate buffer, 6 ml of extraction solvent and 2.5 ml of copper reagent were added. All the tubes were shaken vigorously. 200 mg of activated silicic acid was added and left aside for 30 min. The tubes were centrifuged and 3 ml of the copper layer was transferred to another tube containing 0.5 ml of diphenyl carbazide and mixed carefully. The absorbance was read at 550 nm immediately. The amount of free fatty acids was expressed as mg/100 g tissue.

### Statistical analysis

The data for various biochemical parameters were analysed using analysis of variance (ANOVA) and the group means were compared by Duncan's Multiple Range Test (DMRT). Values were considered statistically significant when at p < 0.05 [31].

## Results

Table 2 shows the level of blood glucose and plasma insulin in control and experimental animals. There was a significant elevation in blood glucose level with significant decrease in plasma insulin levels in alloxan diabetic rats, compared with normal rats. Administration of Diasulin and glibenclamide tended to bring blood glucose and plasma insulin towards near normal levels. The effect of Diasulin at 200 mg /kg was significantly better than 50 and 100 mg/kg, therefore the higher dose was used for further biochemical studies. The administration of Diasulin and glibenclamide to normal rats showed a significant effect in lowering blood glucose and increasing plasma insulin.

**Table 2:** Changes in blood glucose and plasma insulin levels of control and experimental animals

**Groups**	**fasting blood glucose (mg/dL)**	**Plasma insulin (μU / mL)**
Normal	81.58 ± 2.40^a^	11.18 ± 0.74 ^a^
Normal	81.58 ± 2.40^a^	11.18 ± 0.74 ^a^
Diabetic control	265.00 ± 24.40^b^	3.55 ± 0.38 ^c^
Diabetic + Diasulin (50 mg/kg)	210.80 ± 14.26^c^	5.61 ± 0.48^d^
Diabetic + Diasulin (100 mg/kg)	158.33 ± 11.00^d^	6.03 ± 0.41^d^
Diabetic + Diasulin (200 mg/kg)	104.16 ± 6.70^e^	7.05 ± 0.64^e^
Diabetic + Glibenclamide (600 μg/kg)	111.60 ± 9.86^e^	6.32 ± 0.55^de^

Values are given as mean ± S.D for 6 rats in each group.

Values not sharing a common superscript letter differ significantly at p < 0.05 (DMRT).

Duncan procedure, Range for the level 2.89, 3.03, 3.13, 3.20, 3.25.

Diabetic control was compared with normal, ^† ^p < 0.001.

Experimental groups were compared with diabetic control, * p < 0.001.+ + +, > 2% sugar, ++; -2% sugar; +, -1%

Table 3 represents the concentration of TBARS and hydroperoxides in tissues of control and experimental animals. There was a significant elevation in tissue TBARS and hydroperoxides during diabetes, when compared to the corresponding control group. Administration of Diasulin and glibenclamide tends to bring the values to near normal.

**Table 3:** Changes in levels of Tbars and hydroperoxides in liver and kidney of control and experimental animals

**Groups**	**TBARS (mM / 100 g tissue)**		**Hydro peroxide (mM / 100 g tissue)**	
	
	**Liver**	**Kidney**	**Liver**	**Kidney**
Normal	0.73 ± 0.066^a^	0.845 ± 0.07^a^	71.42 ± 4.61^a^	53.56 ± 4.61^a^
Normal + Diasulin (200 mg/ kg)	0.72 ± 0.079^a^	0.785 ± 0.065^a^	69.63 ± 6.09^a^	52.40 ± 3.90^a^
Diabetic control	1.77 ± 0.06^b^	1.565 ± 0.105^b^	102.37 ± 6.41^b^	75.35 ± 4.46^b^
Diabetic + Diasulin (200 mg/kg)	0.88 ± 0.053^c^	0.970 ± 0.072^c^	78.56 ± 5.83^c^	61.89 ± 6.07^c^
Diabetic + Glibenclamide (600 μg/kg)	0.93 ± 0.088^c^	1.00 ± 0.086^c^	80.90 ± 5.32^c^	63.16 ± 6.30^c^

Values are given as mean ± S.D for 6 rats in each group.

Values not sharing a common superscript letter differ significantly at p < 0.05 (DMRT).

Duncan procedure, Range for the level 2.95, 3.09, 3.20.

Table 4 and 5 shows the levels of cholesterol, triglycerides, free fatty acids and phospholipids in liver and kidney of control and experimental rats respectively. Liver and kidney of diabetic rats showed significantly increased levels of cholesterol, triglycerides, free fatty acids and phospholipids, when compared with normal rats. In rats treated with Diasulin and glibenclamide there was a significant decrease in the content of cholesterol, triglycerides, free fatty acids and phospholipids in both the tissues, when compared with diabetic control rats.

**Table 4:** Changes in levels of cholesterol, free fatty acids, triglycerides and phospholipids in liver of control and experimental animals

**Groups**	**Cholesterol (mg/100 g wet tissue)**	**Free fatty acids (mg/100 g wet tissue)**	**Triglycerides (mg/100 g wet tissue)**	**Phospholipids (mg/100 g wet tissue)**
Normal	328.90 ± 18.90^a^	606.10 ± 21.76^a^	344.50 ± 23.20^a^	1598.00 ± 19.30^a^
Normal + Diasulin (200 mg/ kg)	321.25 ± 5.74^a^	601.93 ± 19.00^a^	341.10 ± 21.00^a^	1593.10 ± 24.10^a^
Diabetic control	517.33 ± 13.10^b^	921.60 ± 44.60^b^	622.50± 18.80^b^	1858.60 ± 18.70^b^
Diabetic + Diasulin (200 mg/kg)	421.75 ± 17.54^c^	769.16± 13.30^c^	456.25 ± 17.30^c^	1718.80 ± 14.40^c^
Diabetic + Glibenclamide(600 μg/kg)	445.00 ± 9.79^c^	802.80 ± 53.77^c^	530.80 ± 35.70^d^	1769.30 ± 17.60^c^

Values are given as mean ± S.D for 6 rats in each group.

Values not sharing a common superscript letter differ significantly at p < 0.05 (DMRT).

Duncan procedure, Range for the level 2.95, 3.09, 3.20.

**Table 5:** Changes in levels of cholesterol, free fatty acids, triglycerides and phospholipids in kidney of control and experimental animals

**Groups**	**Cholesterol (mg/100 g wet tissue)**	**Free fatty acids (mg/100 g wet tissue)**	**Triglycerides (mg/100 g wet tissue)**	**Phospholipids (mg/100 g wet tissue)**
Normal	375.90 ± 16.58^a^	434.01 ± 11.50^a^	282.50 ± 14.70^a^	1447.60 ± 26.90^a^
Normal + Diasulin (200 mg/ kg)	371.08 ± 8.28^a^	432.51 ± 11.60^a^	278.75 ± 14.60^a^	1442.50 ± 43.30^a^
Diabetic control	546.90 ± 23.80^b^	743.00 ± 25.70^b^	501.10 ± 34.10^b^	2041.50 ± 33.69^b^
Diabetic + Diasulin (200 mg/kg)	435.20 ± 12.18^c^	556.80 ± 38.50^c^	382.90 ± 9.28^c^	1684.00 ± 28.80^c^
Diabetic + Glibenclamide (600 μg/kg)	449.90 ± 13.49^c^	600.30 ± 33.40^d^	438.66 ± 39.30^d^	1819.30 ± 34.70^d^

Values are given as mean ± S.D for 6 rats in each group.

Values not sharing a common superscript letter differ significantly at p < 0.05 (DMRT).

Duncan procedure, Range for the level 2.95, 3.09, 3.20.

## Discussion

Diabetes mellitus is one of the most common chronic disease and is associated with hyperlipidemia and co-morbidities such as obesity, hypertension. Hyperlipidemia is a metabolic complications of both clinical and experimental diabetes [32]. Alloxan, a beta cytotoxin, induces "chemical diabetes" (alloxan diabetes) in a wide variety of animal species by damaging the insulin secreting pancreatic -cell, resulting in a decrease in endogenous insulin release, which paves the ways for the decreased utilization of glucose by the tissues [33]. In our study, we have observed that Diasulin decreases blood glucose in alloxan diabetic rats. The possible mechanism of action of extract could be correlated with the reminiscent effect of the hypoglycemic sulphonylureas that promote insulin secretion by closure of K^+^-ATP channels, membrane depolarization and stimulation of Ca^2+ ^influx, an initial key step in insulin secretion. In this context, number of other plants has also been reported to have antihyperglycemic and insulin stimulatory effects [34,35]. Like the plant extract, glibenclamide also produced significant reduction in blood glucose levels of alloxan diabetic rats. Since alloxan is known to destroy pancreatic β-cells, the present findings appear to be in consonance with the earlier suggestion of Jackson and Bressler [36] that sulphonylureas have extra- pancreatic antihyperglycemic mechanism of action secondary to their insulin secreting effect and the attendant glucose uptake into, and utilization by, the tissues.

Apart from the regulation of carbohydrate metabolism, insulin also plays an important role in the metabolism of lipids. Insulin is potent inhibitor of lipolysis. Since it inhibits the activity of the hormone sensitive lipases in adipose tissue and suppresses the release of free fatty acids [37]. During diabetes, enhanced activity of this enzyme increases lipolysis and releases more free fatty acids in to the circulation [38]. Increased fatty acids concentration also increases the β-oxidation of fatty acids, producing more acetyl CoA and cholesterol during diabetes.

In normal condition, insulin increases the receptor-mediated removal of LDL-cholesterol and decreased activity of insulin during diabetes causes hypercholestrolemia. Hypercholestrolemia and hypertriglycridemia have been reported to occur in diabetic rats [39]. The increased concentration of cholesterol could result in a relative molecular ordering of the residual phospholipids resulting in a decrease in membrane fluidity [40]. The increased concentration of free fatty acids in liver and kidney may be due to lipid breakdown and this may cause increased generation of NADPH, which results in the activation of NADPH dependent microsomal lipid peroxidation. Liver and kidney phospholipids were increased in diabetic control rats. Phospholipids is present in cell membrane and make up vast majority of the surface lipoprotein forming a lipid bilayer that acts as an interface with both polar plasma environment and non-polar lipoprotein of lipoprotein core [41]. Phospholipids are vital part of biomembrane rich in PUFA, which are susceptible substrate for free radicals such as O_2_^•- ^and OH^• ^radicals [42]. Increased phospholipids levels in tissues were reported by [43,44] in streptozotocin diabetic rats. Administration of Diasulin decreased the levels of tissue free fatty acids and phospholipids.

Accumulation of triglycerides is one of the risk factors in Coronary Heart Disease (CHD). The significant increase in the level of triglycerides in liver and kidney of diabetic control rats may be due to the lack of insulin. Since under normal condition, insulin activates the enzyme lipoprotein lipase and hydrolysis triglycerides [45]. Diasulin reduces triglycerides in tissues of alloxan-induced diabetic rats and may prevent the progression of CHD.

The results show increased lipid peroxidation in the tissues (liver and kidney) of diabetic control group. Previous studies have reported that there was an increased lipid peroxidation in liver, kidney and brain of diabetic rats [46,47]. This may be because the tissues contain relatively high concentration of easily peroxidizable fatty acids. Liver during diabetes, showed a relatively severe impairment in antioxidant capacity than kidney. The kidney exhibits a characteristic pattern of changes during diabetes [48]. The increase in oxygen free radicals in diabetes could be primarily due to increase in blood glucose levels, which upon autoxidation generate free radicals and secondarily due to the effects of diabetogenic agent alloxan [49]. In diabetes, hypoinsulinaemia increases the activity of the enzyme, fatty acyl coenzyme, coenzyme A oxidase, which initiates β-oxidation of fatty acids resulting in lipid peroxidation [50,51] Increased lipid peroxidation impairs membrane functions by decreasing membrane fluidity, and changing the activity of membrane-bound enzymes [51]. Its products (lipid radicals and lipid peroxide) are harmful to the cells in the body and are associated with atherosclerosis and brain damage [51]. Administration of Diasulin and glibenclamide reduced the lipid peroxidative markers in liver and kidney tissues of diabetic rats. This indicates that Diasulin inhibit oxidative damage due to the antiperoxidative effect of ingredients present in Diasulin. This could be correlated with previous study that reported that *Cassia auriculata *[52,53], *Syzigium cumini *[54,55]*Tinospora cardifolia *[56], and *Scoparia dulcis *[57] (ingredients of Diasulin) have antiperoxidative and antihyperlipidemic effect of diabetic animals.

Antidiabetic and antihyperlipidemic effect of Diasulin may be due to the effect of active constituents of different plants, viz, alkaloid and pectins from *Coccinia indica *[58] alkaloids from *Tinospora cordifolia *[59], emlicanin A and B from *Emblica officinalis *[60], trigonelline and scopoltin from *Trigonella foenum graecum *[61], alkaloid-6-methoxybenzoxazolinone and terpenoids such as scoparic acids A,B,C and scopadulcic acid A and B from 'scoparia dulcis' [62], which may be responsible for scavenging free radicals liberated by alloxan in diabetic rats.

On the basis of above results, it could be concluded that Diasulin, a combination of ten herbal plants exert a significant antihyperlipidemic and antiperoxidative effect. This could be due to different types of active principles, each with a single or a diverse range of biological activities, which serves as a good adjuvant in the present armamentarium of antidiabetic drug.

## Conclusion

The liver and kidney exhibits numerous morphological and functional alterations during diabetes. Since both diabetes and hyperlipidemia are considered to be major risk factors for the premature atherosclerosis and essentially all the cholesterol in atherosclerotic plaques is derived from that of circulatory cholesterol. The antihyperlipidemic and antiperoxidative effect of Diasulin in particular could be considered as of possible therapeutic value.

## Competing interests

The author(s) declare that they have no competing interests

## Authors' contributions

LP – supervised the design and co-ordination of the study

RS – Practically conducted the design of the study and drafted the manuscript.

## Pre-publication history

The pre-publication history for this paper can be accessed here:


